# Choroidal detachment and hypotony following selective laser trabeculoplasty: a case report

**DOI:** 10.1186/s12886-023-03033-w

**Published:** 2023-06-16

**Authors:** Woong Hee Kim, Seung Hyen Lee, Jeong Hyun Seo, Eun Hye Jung

**Affiliations:** Department of Ophthalmology, Nowon Eulji University Hospital, Eulji University College of Medicine, 68 Hangulbiseok-Ro, Nowon-Gu, Seoul, 01830 Republic of Korea

**Keywords:** Hypotony, Choroidal detachment, Selective laser trabeculoplasty, Complication

## Abstract

**Background:**

Selective laser trabeculoplasty (SLT) is relatively safe and effective in lowering intraocular pressure (IOP). However, although rare, complications can occur after SLT. This report describes a patient with choroidal detachment due to hypotony following SLT without anterior chamber (AC) inflammation.

**Case presentation:**

A 67-year-old man was referred for elevated IOP in his left eye with advanced glaucomatous visual field loss. He had previously been diagnosed with idiopathic uveitic glaucoma in the left eye, for which he underwent laser iridotomy, trabeculectomy, and cataract surgery. At the first visit, the IOP of his left eye measured by Goldmann tonometry was 28 mmHg despite maximally tolerated medical treatment. SLT was performed in his left eye, resulting in an IOP of 7 mmHg 7 days later. At 3 weeks post-procedure, the patient experienced ocular pain and decreased visual acuity in his left eye. Slit-lamp examination revealed deep anterior chamber depth and no inflammation reaction, but the IOP in his left eye was 4 mmHg, and both fundus and B-scan ultrasonography showed serous choroidal detachment. All anti-glaucoma agents were stopped, and the patient was started on treatment with oral prednisolone and cyclopentolate eye drops. Three weeks later, choroidal detachment had resolved and the IOP in his left eye had stabilized at 8 mmHg. Follow-up 3 months later showed that the IOP in his left eye remained stable.

**Conclusions:**

Choroidal detachment-related hypotony is a rare complication of SLT. This possible complication following SLT should be informed to the patients and considered when performing the procedure.

## Background

Selective laser trabeculoplasty (SLT) is a safe and effective modality for reducing intraocular pressure (IOP). SLT uses laser energy of short pulse duration (3 ns) that is selectively absorbed only by pigmented cells of the trabecular meshwork, thus sparing adjacent cells and tissues from structural damage, such as fibrosis, scarring, and extracellular remodeling [1, 2]. These advantages theoretically allow SLT to be repeated [3, 4]. Recently, several studies suggest that SLT may have potential as a first-line treatment for glaucoma, based on relatively few adverse events and its efficacy is equivalent to that of IOP-lowering eye drops [5, 6]. Although acute IOP spike and inflammation of the anterior segment are frequent side effects of SLT, these are generally mild, transient and resolve spontaneously [7–9]. This report describes a patient who underwent SLT and developed choroidal detachment following hypotony during the early post-procedural period.

## Case presentation

**Fig. 1:**
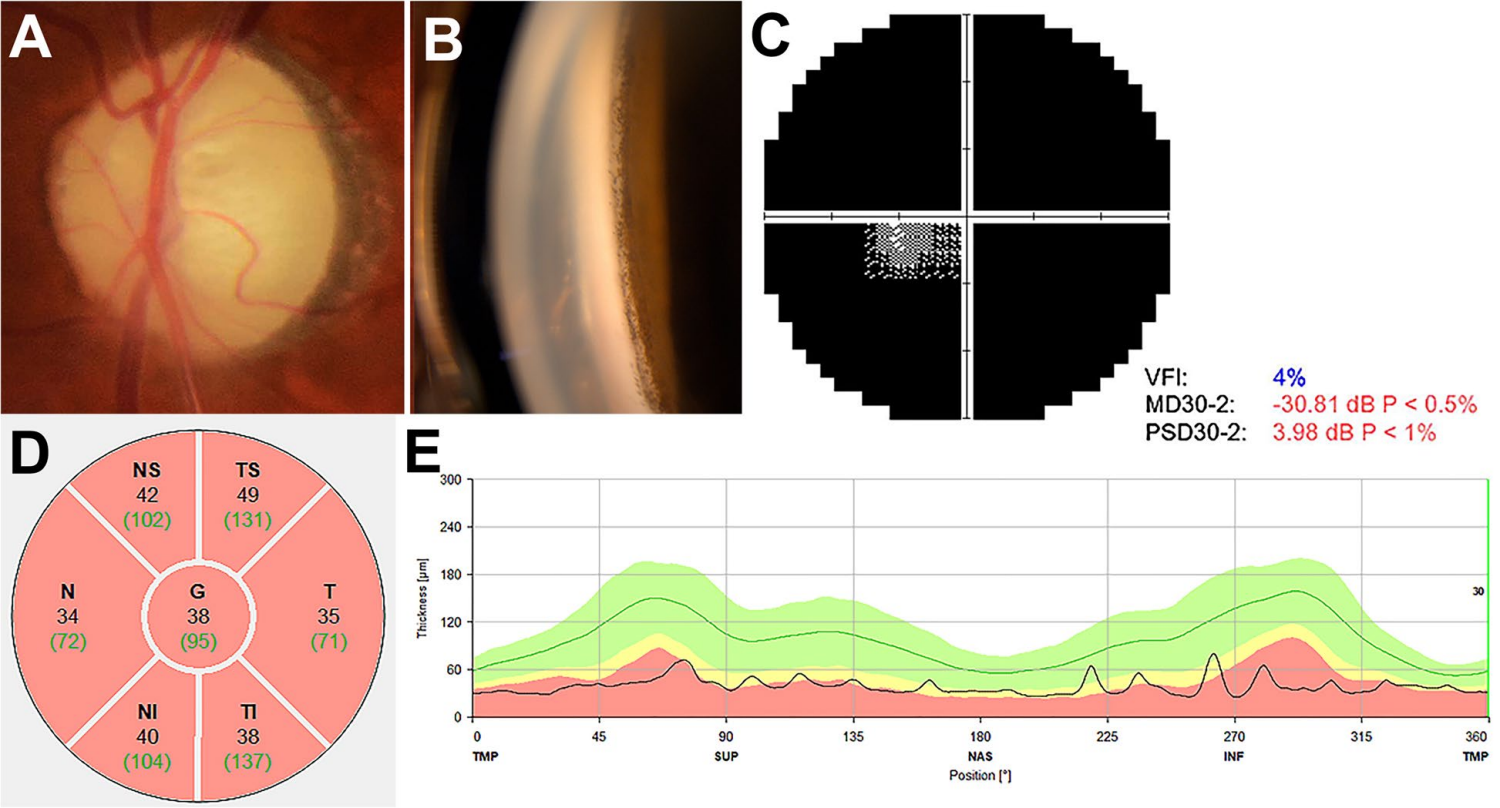
**A** Fundus photograph of the glaucomatous optic disc. **B** Gonioscopic findings, showing an open angle and a hyperpigmented trabecular meshwork. **C** Automated perimetry (Humphrey 30–2) demonstrating severe constriction of the visual field (mean deviation: -30.81 decibel). **D**, **E** Optical coherence tomography of the optic nerve head showing diffuse retinal nerve fiber layer loss

A 67-year-old man was referred for uncontrolled increased IOP in his left eye despite maximal tolerated medical therapy. He had previously been diagnosed with left secondary glaucoma related to idiopathic uveitis, which was diagnosed based on negative results in all systemic work-ups including serologic, radiologic test. His previous ocular history included laser iridotomy (LI) and trabeculectomy, which were performed three years prior to SLT procedure, as well as phacoemulsification with in-the-bag posterior chamber lens implantation which performed two years ago. His past medical history was unremarkable. Best corrected visual acuity was 20/20 in his right eye and 6/20 in his left eye. IOP measured by Goldmann applanation tonometry was 15 mmHg in his right eye and 28 mmHg in his left eye, with the latter being treated with a fixed combination of dorzolamide and timolol (Cosopt, MSD, Switzerland) twice daily, brimonidine 0.2% (Alphagan, Allergan, USA) twice daily and latanoprost 0.005% (Xalatan, Pfizer, Belgium) once nightly. Slit lamp examination showed deep anterior chambers in both eyes, as well as showing a shallow bleb, a patent LI hole at the 11 o’clock position of the iris, and an iridectomy site at the 12 o’clock position of the iris in the left eye. Gonioscopic examination of the left eye revealed peripheral anterior synechiae around the iridectomy site at the 12 o’clock position and hyperpigmented tissue within the trabecular meshwork in all quadrants (Fig. 1B). The patient was prescribed oral acetazolamide (Acetazol tab. 250 mg, Hanlim Pharm., Korea) twice daily for 1 week, reducing the IOP in his left eye to 17 mmHg as measured by Goldmann tonometry. Owing to drowsiness, however, the oral drug had to be discontinued. One week after discontinuing oral acetazolamide, the IOP returned to 30 mmHg. Therefore, it was decided to perform SLT while maintaining twice daily dosing of oral acetazolamide. SLT, rather than additional glaucoma surgery, was selected to reduce IOP because of following findings suggesting advanced glaucoma in the left eye [10], (1) a pale disc on fundus examination, (2) diffuse retinal nerve fiber layer (RNFL) loss on optical coherence tomography (OCT), and (3) severe field constriction on Humphrey visual field test (Fig. 1).

Proparacaine topical anesthetic solution was applied to the left eye, and hydroxypropyl methylcellulose (Hycell solution 2%, Samil Pharm Co., Ltd., Korea) was used with the gonioscopy lens. SLT was performed with 107 shots treating 360° of the trabecular meshwork with 1.1 mJ, which falls within the typical power range of 0.4–1.4 mJ [11]. Thirty minutes after the treatment, the IOP in the left eye was 17 mmHg. The patient was instructed to apply fluorometholone acetate 0.1% (Flarex, Novartis Pharm., Switzerland) drops four times per day for 3 days and to return in a week for follow-up.

**Fig. 2:**
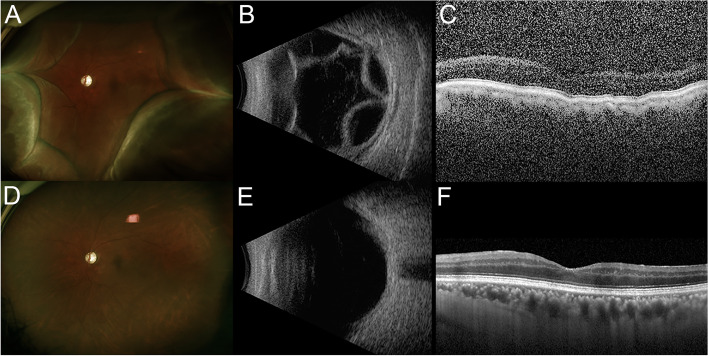
**A-C** Choroidal detachment 21 days after selective laser trabeculoplasty (SLT). **A** Wide-field fundus photography and (**B**) B-scan ultrasonography showing choroidal detachment, accompanied by an intraocular pressure of 4 mmHg. **C** Optical coherence tomography (OCT) demonstrating a chorioretinal fold due to hypotonic maculopathy. **D-F** Improvement of choroidal detachment 2 months after SLT. **D** Wide-fundus photography and (**F**) OCT showing a flat retina without folding. **E** B-scan ultrasonography showing complete resolution of choroidal effusions

One week after the procedure, the IOP in the left eye was 7 mmHg and there were no other symptoms other than foreign body sensation in the eye. Significant anterior chamber shallowing and inflammation were also not observed. Considering that the IOP decreased sufficiently, oral acetazolamide was discontinued and the patient followed-up 2 weeks later. At that visit, the patient reported ocular pain in the left eye when touched. The best corrected visual acuity and IOP of the left eye were 3/20 and 4 mmHg, respectively. Slit lamp examination showed a deep anterior chamber without evidence of marked inflammation. Fundus examination and B-scan ultrasonography revealed hypotonic maculopathy and choroidal detachment in the left eye (Fig. 2A, B, C).

All anti-glaucomatous drugs were discontinued and the patient was started on oral prednisolone acetate (Solondo tab, Yuhanmedica, Korea) 30 mg per day. One week later, the IOP in the left eye was 4 mmHg, and the choroidal detachment did not improve. The patient was therefore started on additional atropine 1% (Isopto atropine, Alcon, USA) twice daily and prednisolone acetate suspension (Pred Forte 1%, Allergan, USA) four times daily. The IOP in his left eye was 7 mmHg 3 days later and 23 mmHg 10 days later, and the choroidal detachment had improved (Fig. 2D, E, F). One week after discontinuation of oral prednisolone acetate, atropine eye drops, and prednisolone acetate suspension, the IOP in his left eye was stable at 8 mmHg. Three months after SLT, the IOP in that eye was well maintained at 10 mmHg, with the patient being treated with only one anti-glaucomatous drug, brimonidine.

## Discussion and conclusions

The present report describes a patient who experienced choroidal detachment accompanied by hypotony following SLT. Two cases of choroidal effusion after SLT have been reported previously, however, these are all related with severe iritis [12, 13]. To our knowledge, this is the first report of hypotony and choroidal detachment without inflammation after SLT.

SLT is regarded as a comparatively safe procedure with few significant side effects. Common complications associated with SLT include conjunctival injection, mild anterior chamber reaction, and post-treatment transient IOP elevation, all which were temporary and resolved with appropriate treatment [7–9]. Other rare reported complications include cornea-related diseases, such as edema, decompensation, hyperopic shift, subsequent corneal thinning, irregular astigmatism, and herpetic stromal keratitis [14–16]. In addition, hyphema [17], hypopyon [18], and cystoid macular edema [19] after SLT have been reported.

The mechanism by which SLT lowers IOP has not been clearly determined. SLT is a laser procedure, in which energy is theoretically absorbed only by the pigmented trabecular meshwork (TM). This selective absorption causes less disruption to the TM than argon laser trabeculoplasty, as demonstrated in previous histopathological studies [2, 20]. These mechanical, structural and biologic alterations increase aqueous outflow through the TM. Inflammatory reaction replaces the extracellular matrix, with TM cells secreting new cytokines. This, in turn, can alter tissue permeability, with cytokine-induced phagocytes thought to remove cell debris and alter aqueous outflow [21, 22].

The mechanism underlying hypotony-associated choroidal detachment in this patient remains unclear. Based on the IOP lowering mechanism of SLT, following three possible mechanisms can be presumed: (1) development of uveal inflammation, (2) increased uveoscleral aqueous outflow associated with structural changes in the ciliary body, and (3) aqueous suppression associated with drug use. SLT selectively affects pigmented TM cells, with gonioscopy showing hyperpigmentation of the TM in this patient. Thus, the TM in this patient may have absorbed excess laser energy, enhancing the inflammatory response. Although evidence of anterior iritis was not observed in this patient, cyclitis and choroiditis may have occurred in the posterior segment of the eye. Inflammation in the posterior segment may have contributed to increased vascular permeability, enabling the transudation of fluid from the choroidal vasculature into the suprachoroidal space [23, 24]. This transudation, in turn, may have increased the fluid content of the choroidal stroma, leading to choroidal detachment. Alternatively, although gonioscopic examination showed no evidence of obvious cyclodialysis or any discernible cleft, multiple operations on this eye may have resulted in subclinical dialysis, thereby affecting uveoscleral outflow [25]. Treatment with oral steroids did not improve the choroidal detachment associated with hypotony, whereas instillation of atropine was effective. Although the choroidal detachment may have improved spontaneously over time, the effectiveness of atropine suggests that the TM did not function properly, rather than that hypotony was caused by an inflammatory reaction. A third possibility was that hypotony was caused by the previously use of anti-glaucomatous eye drops and oral medications. Hypotony has been associated with prior long-term treatment with aqueous suppressants, such as carbonic anhydrase inhibitors. This may result in oversensitivity of ciliary epithelium to subsequent pharmacological therapy [26]. The patient in this study was treated with the same hypotensive eye drops and oral medications before and after SLT, suggesting that choroidal detachment due to hypotony may have resulted from the effect of these drugs.

SLT is generally contraindicated in eyes with secondary glaucoma associated with inflammation [27, 28]. When IOP cannot be controlled in uveitic glaucoma, surgical treatment may be considered. In this case, however, anterior chamber inflammation was not observed during follow-up and additional glaucoma filtration surgery was considered dangerous due to the severity of visual field loss [29]. Moreover, the patient did not want to undergo additional surgery. The benefits and risks of treatment methods were discussed in detail, including the risk of complete sight loss after surgery. The patient and surgeon agreed with performing SLT in the left eye, because there were no other treatment options except for surgery. The patient maintained a stable low IOP three months after the procedure, indicating that SLT can have an IOP lowering effect. Thus, SLT should be considered an alternative to surgery, especially when there are no other treatment options.

In conclusion, SLT should be performed with caution, especially in eyes with a previous history of glaucoma surgery, as SLT can be associated with rare but serious complications, such as hypotony.

## Data Availability

All data generated and analyzed this study are included in this article.
